# Editorial: AI empowered cerebro-cardiovascular health engineering

**DOI:** 10.3389/fphys.2023.1335573

**Published:** 2023-12-12

**Authors:** Lisheng Xu, Zengzhi Guo, Dingchang Zheng, Jianbao Zhang, Fei Chen, Rong Liu, Chunsheng Li, Wenjun Tan

**Affiliations:** ^1^ College of Medicine and Biological Information Engineering, Northeastern University, Shenyang, China; ^2^ Key Laboratory of Medical Image Computing, Ministry of Education, Shenyang, China; ^3^ Research Centre of Intelligent Healthcare, Coventry University, Coventry, United Kingdom; ^4^ Department of Life Science and Technology, Xi’an Jiaotong University, Xi’an, China; ^5^ Department of Electrical and Electronic Engineering, Southern University of Science and Technology, Shenzhen, China; ^6^ School of Biomedical Engineering, Dalian University of Technology, Dalian, China; ^7^ Department of Biomedical Engineering, Shenyang University of Technology, Shenyang, China; ^8^ School of Computer Science and Engineering, Northeastern University, Shenyang, China

**Keywords:** artificial intelligence, neurophysiological signal and image, signal processing, wearable device, cerebro-cardiovascular health, identification and modeling, diagnosis and treatment, rehabilitation

## Introduction

Cerebrovascular and cardiovascular diseases continue to pose significant challenges, contributing significantly to disabilities and mortality rates worldwide (R.L. Sacco et al., 2016). In recent years, artificial intelligence (AI) has emerged as a powerful tool in the realm of cerebrovascular and cardiovascular disease treatment. Its ability to improve decision-making, diagnosis, and treatment processes by analyzing extensive patient data sets has revolutionized medical practices in these fields. This transformative impact is made possible by the rapid advancements in machine learning, deep learning, computational power, and mathematical algorithms, enabling the swift and efficient analysis of vast datasets. Therefore, the integration of AI into cerebrovascular and cardiovascular healthcare has attracted considerable attention among healthcare professionals. Physicians are seeking increasing support from AI tools which improve disease diagnosis, intervention guidance, and therapy monitoring.

This Research Topic “*AI Empowered Cerebro-Cardiovascular Health Engineering*” focuses on recent advancements in AI techniques for enhancing the diagnosis, monitoring, and treatment of cerebrovascular and cardiovascular diseases, including but not limited to noninvasive acquisition methods, accurate diagnosis techniques, personalized therapy approaches, early prediction models, intelligent screening processes, and neurological and cardiovascular rehabilitation strategies. In this Editorial Article, we provide a comprehensive summary of the contributions from the publications within this Research Topic, shedding light on the cutting-edge developments that are shaping the future of cerebrovascular and cardiovascular healthcare through the power of AI.

## Diagnosis of cardiovascular diseases

Screening for cardiovascular disease by the auscultation of heart sound auscultation is a simple, and effective method. Signal processing and AI techniques have been widely used in analyzing heart sounds (Herzig et al., 2014). Li et al. proposed a new heart sound classification method based on improved mel-frequency cepstrum coefficient features and deep residual learning method that achieved an accuracy of 94.4%. This study contributed a more accurate and effective approach to analyzing heart sounds, thus aiding the diagnosis of cardiovascular diseases.

Congenital heart disease (CHD) is one of the leading causes of death (Meras et al., 2021). TrueVue, TrueVue Light, and TrueVue Glass are innovative three-dimensional (3D) echocardiographic techniques that can provide radiation-free visualization of cardiac anatomical structures (Genovese et al., 2019). Sun et al. explored their individual advantages and limitations and summarized their application methods for CHDs. This research demonstrated that the combined use of TrueVue, TrueVue Light, and TrueVue Glass improved the accuracy of CHD evaluation and treatment than the traditional imaging techniques.

Heart failure (HF) results from ventricular filling or ejection dysfunction (Hao et al., 2019). Ballistocardiography (BCG) has been utilized for in-home detection of various cardiac diseases recently (Wen et al., 2019). Feng et al. proposed a machine learning-aided scheme based on BCG signals for HF detection, enabling comprehensive exploration of the relationship between the heart and the lung systems. Experimental results demonstrated that the proposed scheme could significantly improve the accuracy and robustness of in-home HF detection.

Early detection of abnormal electrocardiogram (ECG) patterns is an important method for preventing, identifying, and diagnosing cardiovascular diseases (Dong and Zhu, 2004). Shan et al. proposed an ECG anomaly detection framework (ECG-AAE) based on an adversarial autoencoder and temporal convolutional network. They also employed the adjournment network to solve the ECG data imbalance problem. ECG-AAE could effectively improve the detection rate of abnormal ECG patterns than other popular outlier detection methods while ensuring accuracy.

Coronary artery segmentation is essential for helping doctors to identify and segment the regions of interest. However, automatic segmentation of coronary arteries poses challenges due to issues like over-segmentation or omission, often stemming from the small size and poor distribution of contrast medium (Kroft et al., 2007). Wang et al. proposed a novel automatic method, DR-LCT-UNet, to improve the performance of coronary artery segmentation by preserving unobtrusive features through dense residual connection and focusing on local contextual information. The dice similarity coefficient, Recall, and Precision of DR-LCT-UNet outperformed other comparison methods.

The central aortic pressure waveform (CAPW) contains valuable information about the cardiovascular system, making it useful for predicting and diagnosing cardiovascular diseases (Flores Geronimo et al., 2021). Sun et al. proposed a novel method to approximate the actual flow waveform with a personalized flow waveform and to examine the feasibility of decomposing the CAPW for quantifying wave reflection. This study proposed a more accurate method based on the characteristics of the CAPW to estimate wave reflection indices than the traditional triangular and lognormal flow methods.

## Diagnosis of cerebrovascular diseases

Intracranial pressure (ICP), defined as the pressure within the craniospinal compartment, is an important physiological parameter reflecting the biomechanical status of the brain (Czosnyka and Pickard, 2004). The photoplethysmography (PPG) technology has been applied in the daily monitoring of various physiological parameters. Liu et al. developed a computational model of intracranial PPG signals for the first time and investigated the effect of ICP changes on the waveforms of intracranial PPG signals in different cerebral perfusion territories. This research demonstrated that ICP values could significantly change the value-relevant (maximum, minimum, mean, and amplitude) waveform features of PPG signals measured from different cerebral perfusion territories.

## Treatment of cardiovascular diseases

Functional single ventricle (FSV) is a complex congenital malformation with only one fully functional ventricle. The Y-shaped conduit total cavopulmonary connection (YCPC) was a theoretically efficient procedure for the treatment of FSV. However, the theoretical effectiveness of TCPC significantly differs from its postoperative efficacy (Trusty et al., 2016). To bridge this gap, Zhang et al. applied preoperative computed tomography angiography and computational fluid dynamics simulation to predict and verify the efficacy of YCPC. This approach has effectively improved the applicability of clinical applications of YCPC.

Left atrial appendage occlusion (LAAO) was used as a new approach for stroke prevention in patients with atrial fibrillation (AF) (Turagam et al., 2020). 3D Auto LAA is an AI tool designed for the automatic identification of the LAA and measuring important parameters related to surgery. Sun et al. were the first to combine TrueVue Glass and 3D Auto LAA to evaluate AF patients undergoing LAAO and explore their clinical value and technical advantages. They found that it provided a more accurate and efficient assessment of LAA anatomy and physiological parameters for LAAO, surpassing other commonly used imaging techniques.

Abdominal aortic aneurysm (AAA) is a degenerative disease that causes significant health problems in humans (Kugo et al., 2019). Artemisia annua L. (A. annua) is a traditional herbal remedy that has been widely used in cardiovascular disease. Molecular docking, a statistical simulation method, focuses on the interaction between molecules and predicts their binding mode and affinity (Wang and Zhu, 2016). Jia et al. systematically summarized the molecular targets of A. annua in the treatment of AAA using network pharmacology combined with molecular docking technology. The molecular docking results revealed that the top five active components of A. annua had a good affinity for core disease targets and played a central role in treating AAA. The results showed that the low binding energy molecular docking results provided valuable information for the development of drugs to treat AAA.

## Treatment of neurological diseases

Epilepsy is the most common chronic neurological disease. Intracranial electroencephalography (EEG) signal provides precise anatomical information about the selective engagement of neuronal populations at the millimeter scale and stereo- EEG is one kind of Intracranial EEG (Parvizi and Kastner, 2018). EEG phase-amplitude coupling (PAC), the amplitude of high-frequency oscillations modulated by the phase of low-frequency oscillations (LFOs), is a useful biomarker to localize epileptogenic tissue (Guirgis et al., 2015). Li et al. proposed a novel approach for generating complex-valued PAC with both the coupling strength and the coupled phase of LFO to identify pathological PAC in Stereo-EEG from patients with epilepsy. Their proposed method held promise for identifying more accurate epileptic brain activity for potential surgical intervention. In a separate contribution, Liu and Li proposed a novel approach for localizing neuromodulatory targets, which used intracranial EEG and multi-unit computational models to simulate the dynamic behavior of epileptic networks through external stimulation. This study provided a new tool for localizing patient-specific targets for neuromodulation therapy.

The innervation zone (IZ) of a muscle is the region where muscle fibers are innervated by motor axon terminals. IZ estimation is important for treating patients with neurological injuries such as stroke and cerebral palsy (Zhang et al., 2021). Huang et al. investigated methods to reliably and automatically estimate the IZ from monopolar M-wave recordings and found that the PCA-based method demonstrated the most consistency with manual detectionthan other methods. This study has significant clinical value for the neurological rehabilitation of patients.

## Neurological and cardiovascular rehabilitation

Even after treatment, both cardiovascular and cerebrovascular diseases can result in motor or cognitive dysfunction, requiring a long period of rehabilitation. A rehabilitation process combining motor and cognitive training has the potential to enhance the chance of recovery and rebuild the action ability. 3D gaming and virtual reality have been employed to make the process more immersive (Nasri et al., 2020). Chen et al. proposed an interactive game utilizing real-time skeleton-based hand gesture recognition to assist rehabilitation exercises. This approach improved the hand-eye coordination of the patients during a game-like experience. A lightweight residual graph convolutional architecture was proposed for hand gesture recognition. Most of the participants in this study showed an improvement in their passing rate of the game during the test process.

## Risk prediction of cerebrovascular and cardiovascular diseases

Pre-eclampsia (PE) is a type of hypertensive disorder that can occur during pregnancy (Duhig et al., 2019). Li et al. developed and validated a predictive model for predicting the risk of PE. The model was further analyzed in two subgroups: early-onset pre-eclampsia and late-onset pre-eclampsia at prenatal visits at different gestational weeks. The overall accuracy of the model reached 86%. This predictive model for PE is of great significance in giving targeted clinical predictions and recommendations, ultimately contributing to the improvement of maternal and infant conditions.

Myocardial infarction (MI) is a prevalent cardiovascular disease (R. Nasimov et al., 2020). Zhou et al. developed an algorithm using case data to determine the relationship between the physiological indicators of MI patients and their prognosis. The patient prognostic risk prediction model had an impressive accuracy of 92.2%. The results demonstrated that the prediction model could offer an effective solution for clinical disease prognostic risk assessment, leading to improved clinical outcomes.

In summary, this Research Topic presents the most advancements in AI applications within the fields of cerebrovascular and cardiovascular healthcare. These studies represent a significant stride in our understanding of how AI technology can profoundly improve the diagnosis, treatment, risk prediction, and rehabilitation of cerebrovascular or cardiovascular disease. We appreciate the efforts of all authors for their invaluable contributions, which have immensely enriched this Research Topic. Furthermore, we express our gratitude to the diligent reviewers and editors for their tremendous efforts in ensuring the quality of rigor of the published work. We sincerely hope that readers will find this Research Topic to be both insightful and valuable, offering a comprehensive overview of the transformative potential of AI in revolutionizing cerebrovascular and cardiovascular healthcare.
